# Burnout among resident doctors: An observational study

**DOI:** 10.1016/j.amsu.2022.103437

**Published:** 2022-03-12

**Authors:** Sudha Shahi, Dhundi Raj Paudel, Tika Ram Bhandari

**Affiliations:** a Otorhinolaryngology Head and Neck Surgery, National Academy of Medical Sciences, Bir Hospital, Kathmandu, Nepal; b General Surgery, People's Dental College and Hospital, Kathmandu, Nepal

**Keywords:** Burnout, Resident doctors, Mental health, Residency training, Patient care

## Abstract

**Background:**

Burnout is a syndrome of emotional exhaustion and depersonalization that reduces efficiency at work. No studies have been reported focusing only on residency burnout and risk factors from our country until now. This study aimed to find out the impact and the association of specific demographic and practice characteristics with burnout among resident doctors.

**Methods:**

A prospective cross-sectional survey of all resident doctors under training at that point of time in 2019 in the National Academy of Medical Sciences, Nepal in different specialties was done. We evaluated demographic variables, practice characteristics, and assessed burnout through validated Maslach burnout inventory (MBI) tools, and data were analyzed.

**Results:**

A total 347 among 410 resident doctors (227 male) responded to the survey. Median age was 30 years (range 25–44). Overall, 147 (42.4%) of responding residents were burned out with high emotional exhaustion in 58 (16.6%), high depersonalization in 55 (15.9%), and low personal achievement in 34 (9.8). In regression analysis, out of independent variables gender, marital status, having children, specialty, hours of work per week and year of residency, specialties (general surgery odds ratio [OR]; 12.595, confidence interval [CI],[ 1.037–152.9], P; 0.047), obstetrics, and gynecology (odds ratio [OR]; 13.977, confidence interval [CI]; [1.324–147.5], P; 0.028), and anesthesiology (odds ratio [OR]; 11.54, confidence interval [CI]; [1.014–131.4], P; 0.049)) and hours of work per week (≥80 h) (odds ratio [OR]; 2.511, confidence interval [CI]; [1.128–5.589], P; 0.024), were significantly associated with high burnout.

**Conclusions:**

Burnout is common among trainee resident doctors which is possibly preventable. Thus, the concern should be to prepare strategies to identify and minimize burnout from the individual, institutional, and societal sides. It is essential to preserve and promote the mental health of trainee residents to prevent serious consequences in the personal lives of resident doctors and as well as on patient outcomes.

## Highlights

• Burnout amongst resident doctors is extremely predominant which may be caused due to various reasons.
• We describe the impact of burnout among resident doctors and focus on identifying different risk factors for burnout.
• Preservation of mental health of resident doctors is essential to prevent serious consequences in their lives as well as on patient care.

## 1 Introduction

Burnout is a syndrome consisting of a triad of emotional exhaustion (emotional overextension and exhaustion), feeling of depersonalization (negative, callous, and detached responses to others), and reduced personal accomplishment mainly to one's professional work. A validated assessment tool frequently used to evaluate burnout in medical professionals is Maslach Burnout Inventory–Human Services Survey (MBI) [1].

Residency is a challenging period in doctor training in all specialties. Many resident doctors during their training experience different levels of stress and this continuous pressure can cause feelings of discouragement and dissatisfaction, which might lead to the desire of leaving a training program. Adjusting to these hectic job burdens affects individual emotional and intellectual contingency, and the capacity to create a healthy home-work balance. The prevalence of burnout among residents diverges widely from 3% to 88% [2]. Burnout in residents can have serious effects not only on personal well-being, interpersonal relationships as well as on career but also may have negative effects on patient care. For the sustainability of health care systems, the well-being of residents is vital. A good understanding of factors that are associated with burnout in residents has important significance [3]. There are growing global concerns regarding stress in the professional and personal lives of residents leading to the increased focus on their well-being [4].

This study aimed to identify the impact of burnout in Nepalese resident doctors and provide a brief overview of the current impact of burnout, factors that contribute to burnout and suggestions for interventions to decrease burnout and minimizing grave consequences to the residents. We believe it can also encourage the concerned authorities in the institution to look into the matter with utmost care so that the institution can acquire more productive results from the resident doctors for the excellence of the institution.

## 2 Materials and method

This is a cross-sectional survey of all resident doctors under training at that point of time in 2019 in the National Academy of Medical Sciences, Nepal. We included all the resident doctors under different specialties under training who gave consent to participate but we excluded resident doctors not willing to participate. The study was approved by the Institutional Review Committee of the National Academy of Medical Sciences, Bir Hospital, Kathmandu, Nepal. In addition to that, all methods were performed in accordance with the relevant guidelines and regulations. We surveyed using the Maslach Burnout Inventory (MBI) which is a previously validated tool that measures burnout. We distributed demographic survey performa with MBI questionnaire to each resident and response were anonymous and collected. MBI tool consists of a 22-item self-administered 7-point Likert scale questionnaire organized into the following 3 subscales: i) 9 questions on Emotional Exhaustion, ii) 5 questions on Depersonalization, and iii) 8 questions on Personal Accomplishment [1]. This inventory was intended for and validated amid human service workers, comprising residents. Each dimension gets an MBI subscore, that, compared to normal scores for the work population, is classified as low, medium, or high. We considered a high subscore in emotional exhaustion and depersonalization and a lower subscore on personal achievement to define clinically significant burnout similarly mentioned in the literature [5].

The Shapiro–Wilk test was used to test the normality of the data. All continuous variables were compared with an independent *t*-test. The Chi-square test was used for categorical values. Statistical software SPSS version 25.0 (Statistical Package for the Social Sciences) was used for statistical analysis. A p-value < 0.05 was considered statistically significant.

Our work is fully compliant with the STROCSS criteria www.strocssguideline.com in which a completed STROCSS checklist stating the page numbers [6].

## 3 Results

A total of 347 in training resident doctors out of 410 (84.6%) responded to the survey. The median age was 30 years. The majority of residents were male 227 (65.4%) with the male is to female ratio 1.89:1. All participants data were shown in Table 1. There were no significant differences in different variables except gender (male), marital status (unmarried), year of residency, and specialty (P < 0.05) when we compared these variables in burnout and without burnout groups (Table 1).

**Table 1:** Participant's data.

Variable	Burnout n = 147 (42.4%)	Without Burnout n = 200 (57.6%)	P value
Age, (years)	30 (25–38)	30 (25–44)	0.621
Age, (years)			0.660
≥30	84 (57.1)	119(59.5)	
<30	63 (42.9)	81 (42.4)	
Sex			0.025
Male	106 (72.1)	121(60.5)	
female	41 (27.9)	79(39.5)	
Marital Status			0.008*
Unmarried	87(59.2)	87 (43.5)	
Married	58 (39.5)	112 (56.0)	
Divorced	2 (1.4)	1(0.5)	
Having Children			0.904
Yes	25 (17.0)	35(17.5)	
No	122 (83.0)	165(82.5)	
Substance^♣^ abuse			0.500
Yes	63(42.9)	93 (46.5)	
No	84(57.1)	107(53.5)	
Year of residency			0.003*
I	42 (28.6)	81 (40.5)	
II	68 (46.3)	57(28.5)	
III	37 (25.2)	62 (31.0)	
Specialty			0.001*
Internal Medicine	12 (8.2)	29 (14.5)	
General Surgery	23 (15.6)	5 (2.5)	
Obstetrics & Gynecology	37 (25.2)	10 (5.0)	
Pediatrics	10 (6.8)	24 (12.0)	
ENT^♦^	4 (2.7)	12(6.0)	
Orthopedics	11 (7.5)	17 (8.5)	
Radiology	1 (0.7)	13 (6.5)	
Oncology	0 (0)	3 (1.5)	
Anesthesiology	28 (19.0)	7 (3.5)	
Ophthalmology	10(6.8)	40 (20)	
Dental	0 (0)	6 (3.0)	
Dermatilogy	2(1.4)	6(3.0)	
Pathology	1 (0.7)	4 (2.0)	
MDGP^¶^	7(4.8)	17(8.5)	
Psychiatry	1(0.7)	7(3.5)	
Hours of duty per week			0.001*
≥80	105(71.4)	48(24.0)	
<80	42(28.6)	152(76.0)	

Categorical variables are presented as n (%); Continuous variables are presented as median, ^♦^ENT; ear, nose and throat, ^♣^Substance; smoking, alcohol, caffeine or in combination, ^¶^MDGP; MD in general practice and emergency medicine, *P; value is significant if < 0.05.

When we collected MBI Score for burnout, we found 147 (42.4) high scores followed by moderate 59 (17.0%) and 141 (40.6%) low scores as shown in Table 2. Within the high score, high emotional exhaustion was noted in 58 (16.7%); high depersonalization was in 55 (15.9%) and very low personal achievement in 34 (9.8%) (Table 2).

**Table 2:** MBI score of Burnout.

Components	High^a^ n = 147 (42.4%)	Moderate^b^ n = 59 (17%)	Low^c^ n = 141 (40.6%)
Emotional Exhaustion (EE)	58 (16.7)	27 (7.8)	69 (19.9)
Depersonalization (Dep)	55 (15.9)	18 (5.2)	45 (13.0)
Low Personal Achievement (PA)	34 (9.8)	13 (3.7)	28 (8.1)

a High; EE > 27, Dep>13 and Low PA >39.

b Moderate; EE [[17], [18], [19], [20], [21], [22], [23], [24], [25], [26]], Dep [[7], [8], [9], [10], [11], [12]] and Low PA (32–38), and.

c Low; EE (0–16), Dep (0–6) and Low PA (0–31).

We had also evaluated different factors associated with burnout. In logistic regression analysis, on multivariate analysis, out of independent variables only specialty (general surgery, obstetrics and gynecology, and anesthesiology) and hours of duty per week were found to be significantly associated with an increased risk of burnout (P < 0.05) which is shown in Table 3.

**Table 3:** Association of variables with residents Burnout.

Variables	Burn out n = 147 (42.4%)	Univariate analysis (Chi-Square value); P-value	Multivariate analysis OR;(CI); P-value
Sex (Male)	106 (72.1)	(5.047); 0.025*	1.526; (0.778–2.995); 0.219
Marital status (Unmarried)	87(59.2)	(7.814); 0.005*	2.511; (0.891–2.662); 1.540
Year of residency I II	42 (28.6) 68 (46.3)	(11.828); 0.003*	0.659; (0.330–1.313); 0.236 1.686; (0.850–3.342); 0.135
Specialty General Surgery Obstetrics & Gynecology Anesthesiology	23 (15.6) 37 (25.2) 28 (19.0)	(101.81); 0.001*	12.595;(1.037–152.9); 0.047* 13.977; (1.324–147.5); 0.028* 11.54; (1.014-131.4);0.049*
Hours of work per week (≥80)	105(71.4)	(77.31); 0.001*	2.511; (1.128–5.589); 0.024*

OR: odds ratio; CI: confidence interval; *P; value is significant if < 0.05.

## 4 Clinical discussion

Structured residency programs began in Nepal in the early 1980s with a training period of three years for all specialities. There are currently more than 20 institutions, including both private and government, providing residency training all over the country. Undoubtedly, residents are the backbone of academic health care institutions. Thus to maintain harmony in the institution it is essential to take care of resident's well-being. This is equally important to impart better patients care. While residency burnout has gained international attention, different aspects contributing to burnout have been studied in different parts of the world. However, there is sparse data in our context in this regard with few studies with a generalized approach to burnout and anxiety problems among physicians and medical students but there have been a few studies so far focusing on burnout during residency training [7].

Burnout amongst resident doctors is extremely predominant that may be due to long working hours, high educational demands, lack of autonomy, a high level of work-home interference, a shortage of benefits, and insecurity about the future. Notably, burnout is associated with an intensification of medical errors and ultimately reduced quality of patient care. Adding to it, another study reported that burnout is also associated with absenteeism, substance abuse, and mood disorders [8]. Long work hours are only one aspect contributing to resident burnout. During the passage of their training, residents experience a reduction in sleep hours, improper diet, exercise, family and social interactions, and attendance at special events. More specifically, sleep deprivation has been related to the onset of depression, and the stress of training has even been linked to many resident suicides [9].

A prevalence of burnout reported widely from 3% to 88% in a varied range of medical specialties [2]. Meanwhile, in our study, we found burnout in 147 (42.4%) residents comprising high emotional exhaustion in 58 (16.7%), high depersonalization in 55 (15.9%), and very low personal achievement in 34(9.8) which is comparable with existing literature. Our burnout rate in the residents is higher than that previously reported (28%) in some studies, which may be due to the long working hours and other factors more specific to our context [10].

We had also evaluated different factors associated with burnout among residents and out of different independent variables, we noted that certain specialties (surgery, obstetrics and gynecology, and anesthesiology) and long hours of work per week (more than 80 h per weeks) are significantly associated with an increased risk of burn out. We did not observe any association between marital status, having children, and year of residency between the groups. However, Shanafelt et al. reported in their study that burn out is quite common in younger surgeon and a surgeon having children [11].

On the other hand, many studies have mentioned an inverse correlation between physician age and burnout [11]. These attributable factors could be, survival bias. It usual for young resident doctors who suffered from burnout to be more prone to change careers, while older doctors who remain in their field are more resistant and thus less prone to burnout. However, we did not find out any association of age and level of burnout in our study similar to described in a few studies [12].

Regarding gender associated with burnout, we could not identify any link between gender and burn out similar to Ahmadiyehet al who found that male and female practicing surgeons were equally satisfied with their surgical careers [13]. However, Dyrbye et al. reported women to be significantly more likely to experience burnout and depressive symptoms. Work-home conflicts and hours worked per week have been identified as potential factors that increase burnout among female physicians [14]. Differing to the above-mentioned findings, other studies have denied as women have a higher likelihood of burnout [15]. Remarkably, another study reported that male surgeons are more prone to have burnout [16].

Concerning work hours, many studies found that hours worked per week were a statistically significant predictor of burnout, physical and mental morbidity, decreasing career satisfaction, and decreased work-life balance [17]. Supporting the results above, our residents who worked more than 80 h per week were found to have high odds of suffering from burnout. However, few other studies did not find any significant association between work hours and burnout [18].

Relating to specialty, there are varied results from different studies. We found that general surgery, obstetric and gynecology, and anesthesiology are more troublesome for many residents and are associated with burn out. While few other studies stated that residents with surgical training like otolaryngology (ear, nose, and throat), obstetrics and gynecology, orthopedic surgery, and general surgery were found to have a high risk for burnout [[18], [19], [20]]. However, Doolittle et al. and Gordan et al., in their study mentioned that internal medicine and pediatrics residents respectively have a high risk of burnout [10,21].

Based on the evidence provided by literature, many interventions have been studied for minimizing the impact of resident burnout. They include restriction of duty hours, incorporation of residents wellness program, mindfulness training, the respiratory one method of relaxation, self-development groups, and the 2003 ACGME duty-hour limitations all had one or more studies demonstrating benefit in burnout [22].

The focus has been on resident duty hour restrictions and modification that has gained international attention over the last several decades. Work hour restrictions were initiated to help improve both patient safety and resident well-being [23]. Current American guidelines follow policies set by the Accreditation Council for Graduate Medical Education (ACGME). New requirements have been released in 2017 in which the maximum continuous hours of work are 24 h for all residency programs except emergency medicine which is limited to 12 h [24]. The European Work Time Directive (EWTD) became law in 1998 but only in the last decade been implemented to reduce doctors’ work hours. The current guideline restricts working hours to 48 h per week [25]. In contrast to the European and American duty hour rules, there are no national duty hour restrictions in Canada and many Asian countries like ours [26]. Remarkably, focusing on duty hours alone did not result in improvements in patient care or resident well-being and may have harmed resident education reported recently [27]. While work hour limits appear to be effective in reducing burnout rates among resident physicians, some fear that this might lead to inadequate training of physicians or the lengthening of an already long training period [28].

Alternatively, an emerging solution may be the implementation of resident wellness programs (RWP) into residency training that is considered as new hope and an effort to address burnout as an expansion beyond duty hours regulations. Such programs are defined by a combination of active and passive initiatives targeting the various domains of physical, mental, socioeconomic, and intellectual wellness [9]. The key components of a resident wellness program are; first, providing a safe and confidential environment for residents to express their grievances and motivation for change can be discussed; second, burnout prevention via ongoing surveillance of residents via regular, one-on-one meetings with residents to uncover symptoms of depression, burnout, or substance use; third, employing lectures, workshops to educate residents about burnout and the habits of wellness while discussing all the aspects of trouble faced by residents during their training. Fourth are initiatives that considered physical, mental, social, intellectual, and community wellness that can be accomplished by social outings, gym access, retreats, social outings, mentoring, and charitable contributions. About the efficacy of resident's wellness programs, evidence shows that residents are highly receptive to wellness curricula and participation in wellness sessions has been correlated with higher empathy, reduced anxiety, depression, and ultimately, reductions in burnout scores [9].

Regarding our context, until now we have no strong evidence as to how resident doctors are working under difficult circumstances with minimal facilities, lack of support programs, and unregulated duty hours which are usually defined independently by the institutions depending upon the number of trainees. Thus, we believe this study can give some basis to concerned authorities to take required actions to identify such factors and prepare an action plan for addressing different issues from the institutional level to prevent burnout among resident doctors. There should be guidelines to regulate the total number of work hours per week, salary, wellness support programs, solving problems of lodging, and other reimbursements and rewards for work for the residents. This should be borne in mind that residents are not equivalent to medical students rather are on job medical trainees.

We believe that this is amongst the first study that clearly describes the impact of burnout particularly among resident doctors in our context and that this shall help to advocate for the well being of resident doctors in the training program to yield maximum efficiency. Our response rate (84.6%) was quite satisfying as a comparison to other studies. Lastly, we also acknowledge our limitations that this is single-center study, and being a cross-sectional study, the causal association could not be removed. Moreover, factors such as recall bias and social desirability might faulty come across due to the use of a self-reported questionnaire. Thus, larger prospective multicenter studies would be essential to validate our findings in determining the extent of prevalence of burnout in our settings in the future.

## 5 Conclusions

Burnout is common among trainee resident doctors which is possibly preventable. Thus, the concern should be to prepare strategies to identify and minimize burnout from the individual, institutional, and societal sides. It is essential to preserve and promote the mental health of trainee residents to prevent serious consequences in the personal lives of residents and as well as on patient outcomes.

## Funding

There are no sponsors involved in the study.

## Authors’ contributions

All authors equally contributed to the study concept or design, data searching, data analysis or interpretation, writing the paper.

## Consent

Written and verbal consent has been taken from all the participants for participation and publication.

## Ethical approval

The study was approved by the Institutional Review Committee of National Academy of Medical Sciences, Bir Hospital, Kathmandu, Nepal (705/March 19, 2019).

## Registration of research studies

1. Name of the registry: http://www.researchregistry.com
2. Unique Identifying number or registration ID: 7499
3. Hyperlink to your specific registration: https://www.researchregistry.com/browse-the-registry#home/

## Guarantor

Dr. Sudha Shahi.

## Provenance and peer review

Not commissioned, externally peer-reviewed.

## Declaration of competing interest

All authors declare that they have no competing interests.
